# Supplementary material for: The experience of being a caregiver of patients with digestive cancer, from patients and caregivers’ perception: A mixed study
Source: PLoS One. 2023 Jul 21;18(7):e0287335. doi: 10.1371/journal.pone.0287335 (PMC10361535; doi:10.1371/journal.pone.0287335)
Supplement: S1 File — (DOCX) [file pone.0287335.s001.docx]

**SUPPLEMENTARY FILE 1: Interview guides**

**Supplementary Fila 1.a. Caregiver’s interview guide**

**Caregiver’s interview guide**

1. Tell me a little about yourself

a. How are you feeling right now?

b. How are you doing physically?

c. How is it going morally?

2. Can you tell me how you feel about your role as a caregiver?

a. Tell me about what has changed in your daily life since you took on this new role

b. Tell me about the difficulties you face when helping ……………. (patient’s name)

i. How do you manage these difficulties?

ii. Do you feel you have special needs or require assistance at any level?

c. Given your experience, can you think of any positive experience(s) that might have emerged from this role?

3. How do you think this new role has changed your relationship?

a. Explain to me how you discuss your role together?

b. How do you think your loved one perceives your role as caregiver?

4. How has this changed your view of the future?

a. What are your fears?

i. How do you see the patient dying? And have you ever talked about it together?

ii. About you? Your future?

5. Is there anything else you would like to discuss?

**Supplementary Fila 1.b. Patient’s interview guide**

**Patient’s interview guide**

1. Tell me a little about your caregiver

a. How do you feel he/she is feeling right now?

b. How do you feel he/she is feeling physically?

c. How do you feel he/she is feeling morally?

2. How do you think he/she is experiencing this new caregiving role?

a. How do you feel his/her daily life has been impacted by this new role?

b. What difficulties do you think he/she may encounter?

i. How do you feel he/she is managing these difficulties?

ii. Do you feel that he/she has special needs or requires assistance in certain areas?

c. Given your experience, can you think of any positive experience(s) for him/her that might have emerged from this role?

3. In your opinion, how has the fact that he/she has become your caregiver changed your relationship?

a. Explain how you discuss his/her new role together?

b. Tell me how you perceive this role?

4. How do you think it has changed his vision for the future?

a. What are your fears?

i. What about him/ her?

5. Is there anything else you would like to discuss?
