# Supplementary material for: The experience of being a caregiver of patients with digestive cancer, from patients and caregivers’ perception: A mixed study
Source: PLoS One. 2023 Jul 21;18(7):e0287335. doi: 10.1371/journal.pone.0287335 (PMC10361535; doi:10.1371/journal.pone.0287335)
Supplement: S2 File — (DOCX) [file pone.0287335.s002.docx]

**Supplementary File 2: Additional quotes**

| **Table 1.**  Themes and sub-themes from caregivers’ interviews: additional quotes | |
| --- | --- |
| **THEME 1: Illness is an upheaval in the life of the caregiver** | |
|  | **He must change his habits and re-plan the future**  *“Everything has changed … So first I go to work, I work in, let's say 30 hours. Then I go home, I have to wash the dishes, I have to see if my brother has eaten. I have to wash my own clothes, clean my room. And then sometimes I see my brother's room a little bit, I see if my mom is okay, I check if she ate. Then I have to go shopping. And then I have to think about myself. I have to do my hair and so on. I hope that after that it will change because I can't manage everything at the same time… My time goes by too fast.” – C54*  *“you have to do administrative procedures, files. Send back, start again. What a waste of time!” – C25*  *“I think I'm going to be unemployed pretty soon. And I'm going to try to see if I can't start my own business and see if I can find a job that can reconcile the two.” “It's true that it puts in perspective our projects and we don't know, we don't know how the disease will evolve... The future... The projects are questioned.” – C33*  *“Financially yes, because [the patient] was supposed to go back to work at the end of the year. But she didn’t, so now, inevitably, it puts a strain on the budget, which is quite limited.” “I was off work in November for the time.... The time that I take care of [the patient] and that I make ... that I begin to make my organization.” – C15*  *“On daily life, the disease makes us project ourselves completely differently because we are unable to project ourselves to a month. We don't know. Today, we live from day to day.” – C12*  *“I live with her now, I moved in with her” – C51* |
|  | **because his life is now centered on the illness of his loved one**  *“I would almost call it a full-time job.” – C33*  *“We do according to the disease.” – C53*  *“When she was hospitalized, I was in the hospital every day.” – C51* |
|  | **and it weighs on him mentally and physically.**  *“I am much sadder” – C06*  *“So, after I started to ... to do everything, it's sure that it puts ... it puts, an extra weight and pressure.” – C15*  *“Frankly, I don't sleep. At least compared to before. It's the first time in my life that I see myself so dark, with dark circles like this. Frankly, I'm too tired. Yeah, I'm too tired.” – C54*  *“It affects my sleep” “It is inevitably an additional burden, a moral burden I think” – C01* |
| **THEME 2: He feels alone and helpless dealing with the disease and suffers its consequences** | |
|  | **He has no control over the situation and does not know how to (re)act**  *“It's hard to see him suffer, to see him not being able to eat or do simple things, things he used to do on his own ... That's what I think is the biggest difficulty ...” – C26*  *“Sometimes I think "you're overdoing it”, “you should be nicer, maybe nicer, more this, more that” – C06*  *“Suffering is always the side that is difficult for the patient, but for the people around him too... nothing can be done.” – C25* |
|  |  |
|  | **He dreads what may happen**  *“I wonder... I wonder how it will end... But I don't want to think about it, probably wrongly, but I don't want to think about it... I'm afraid, I can't admit to being alone... It scares me, we have always been together. So, I know that we are not eternal, but I push away this idea... I don't see myself staying alone.” – C26*  *“In fact, our main fear, well my main fear, is the announcement of a fatal end.” – C12* |
|  | **and does not feel heard** |
|  | **He sometimes sees his relationship with the patient deteriorate**  *“We have moved away from each other” – C15*  *“Obviously, but I knew it before, chemo has an impact on our sexuality” – C25* |
| **THEME 3: Despite this, he naturally assumes his role while managing to remain positive** | |
|  | **He acts without asking too many questions**  *“Well, that's the game... How do I feel about it? Well, that's my role. We are married, so it's like they say: "it's for better or for worse". It's challenges that you have to go through together ... challenges are to be gone through together otherwise it's not worth being in a couple.” – C33*  *“No, I don't feel obliged, if it was an obligation, I would have already returned to Gabon...When you do something or help someone, you have to do it with your heart, otherwise it's useless, it's a waste of time.” – C51*  *“He may feel like a burden to me, but that's my role, that's my role ... I'd rather have him at home with me, than in the hospital.” – C26* |
|  |  |
|  | **while managing to draw positive dimensions from it**  *“Beyond the disease, which is still something that is serious ... but it has a positive impact. It permits to sit back and say to yourself: we only have one life on this earth and we have to take advantage of it and stop worrying about things that bother us every day... So it allowed me to take a step back and tell myself that despite the disease, we are happy.” – C12* |
|  |  |

| **Table 2.** Themes and sub-themes from patients’ interviews: additional quotes | |
| --- | --- |
| **Theme 1: The patient sees the caregiver take on his new role naturally and becoming closer to him** | |
|  | **He sees the caregiver helping without asking too many questions**  *“It is not an additional burden for her, on the contrary, it’s what’s she wants.” – P12*  *“She has always preferred to give priority to others, to children, to friends, to people who needed help.” – P06*  *“For him, doing all this is not helping me, it is also his role in the house, he does not take it as a chore” – P15* |
|  | **And being closer**  *“It continued to strengthen our bond even though it wasn't stretched at all... it might sound weird, but I think we're still in love...” – P25* |
| **Theme 2: The caregiver becomes the anchor of the patient** | |
|  | **He sees himself becoming the center of attention of the caregiver**  *“I know that for him, it is not conceivable, for example, not to take me in the morning to the chemo and to come to find me afterwards” – P33*  *“Sometimes at night she has to come in and check on me, and so she doesn't sleep. I started to notice that” – P54*  *“Well, she is constantly worried about me, she is afraid...always saying "but if something happens to you” – P53* |
|  | **The patient recognizes needing him at all times**  *“He does everything, he does everything. He does the cleaning, he does the ironing, he does the shopping, he does the cooking. He does, he does everything, so I'm not saying I don't do anything, but if I really don't have the strength, well, I know I can count on him. That's very important, in terms of daily life” – P33*  *“The two or three times I was able to break down, it was in her company... she reassured me, she told me things, things you need to hear.” – P12* |
|  | **So much so that doesn’t know how he would do without him**  *“She knows, she knows, I think, I hope, all the good I think of her in this regard. And all the thanks I owe her.” – P26*  *“I think he does very well with everything he has to do … I don't know how he does it.” – P25*  *“I’m glad I have my daughter” – P54* |
| **Theme 3: But he notices that this disrupts the life of the caregiver** | |
|  | **He witnesses the burden that this attention generates for the caregiver**  *“At first it was too complicated, it became too complicated for him... it became too complicated to manage everything. He also had to deal with it. So, he took time off work... because he was taking care of everything.*  *“It must be quite heavy for her too, that's for sure. Especially on a moral level” – P01*  *“He had a burn out.” – P33*  *“She manages but it tires her a lot. It anguishes her, but she manages.” – P06*  *“I think it's hard for her, in her role, because she has tasks at home that we used to share together. And now they are more frequent for her than for me, because the week after chemo, well I don't do much, I don't cook, I don't clean, I don't do much, because physically speaking I can't and mentally I don't feel like it and I can't.” – P12* |
|  | **He sees that the caregiver is trying to keep up appearances with him**  *“You can see it, she doesn't show it, she doesn't shout it everywhere but of course she is anxious” – P06* |
| **THEME 4: And this worries him, he feels guilty towards the caregiver and would like to protect him** | |
|  | **He is concerned about the caregiver**  *“My death for example. This will shock her a lot. And it will change her life, that's clear. And that worries me a lot... even if she will be helped by the children, they won't be there all the time.” – P06* |
|  | **And he feels guilty**  *“Well, I always felt like I was in her way” “I always had the impression that I was just there to embarrass them, and that's all...” “I have always felt guilty about my children.” – P01*  *“She dropped out of school. That's what hurts me the most. Because she had, she had her baccalaureate with honors. I feel guilty of that.” – P54*  *“Taking care of me is a role that is... that takes time, she would probably prefer to do something else in life.” – P06* |
|  | **He would prefer that the caregiver accept to be helped** |
|  | **So he tries to protect him in his way**  *“We taught him to ask for help around him. So, I have my mom who offered to do all the laundry for him once a week, so that he could feel a little bit better.” – P15*  *“I try to protect her" – P12* |
